# Sex-specific disparities in disease activity scores among patients with axial spondyloarthritis and their implications for evaluating the response to tumor necrosis factor alpha inhibitor therapy

**DOI:** 10.1186/s13075-024-03320-x

**Published:** 2024-04-25

**Authors:** Seulkee Lee, Seonyoung Kang, Hyungjin Kim, Jaejoon Lee, Min-Ji Kim, Hoon-Suk Cha

**Affiliations:** 1 Department of Medicine, Samsung Medical Center, Sungkyunkwan University School of Medicine, 81 Irwon-ro, Gangnam-gu, Seoul, 06351 Republic of Korea; 2 Biomedical Statistics Center, Research Institute for Future Medicine, Samsung Medical Center, Seoul, Republic of Korea

**Keywords:** Axial spondyloarthritis, Patient reporting outcome, Sex, TNF alpha inhibitor, Disease activity

## Abstract

**Background:**

We aimed to investigate whether there are sex differences in disease activity measures among patients with axial spondyloarthritis (axSpA) and to determine any potential impact on the assessment of treatment responses to tumor necrosis factor alpha inhibitors (TNFi).

**Methods:**

Using the Korean College of Rheumatology Biologics and Targeted Therapy (KOBIO) registry data, we compared sex differences in changes in the Bath Ankylosing Spondylitis Disease Activity Score (BASDAI) and Ankylosing Spondylitis Disease Activity Score (ASDAS) levels at baseline and one year after TNFi initiation in patients with axSpA.

**Results:**

This study included 1,753 patients with axSpA who started or changed TNFi, of whom 1,343 (76.6%) were male. At baseline, the mean BASDAI and ASDAS scores of all patients were 5.98 and 3.6, respectively. The BASDAI changes between baseline and the one-year follow-up were independently associated with sex (𝛽 = 0.343, *p* = 0.011), whereas ASDAS was not (𝛽 = 0.079, *p* = 0.235). When judging the effect of TNFi at one-year of treatment, male patients were more likely to be assessed as effective by the BASDAI-based criterion (ΔBASDAI ≥ 50% or ≥ 2; OR 1.700, 95% CI 1.200–2.406), while the ASDAS-based criterion (ΔASDAS ≥ 1.1) showed no significant difference between sexes (OR 0.993, 95% CI 0.678–1.455), after adjusting for other baseline characteristics.

**Conclusions:**

The changes in disease activity before and after TNFi use were significantly different between sexes when measured by BASDAI, but not ASDAS. TNFi treatment effects may be interpreted differently between sexes depending on the disease activity measure used.

**Supplementary Information:**

The online version contains supplementary material available at 10.1186/s13075-024-03320-x.

## Background

Axial spondyloarthritis (axSpA) is a chronic inflammatory disease predominantly affecting the axial skeleton [1–4]. Radiographic axSpA, also known as ankylosing spondylitis (AS), is more prevalent in males. Since the introduction of the Assessment in Spondyloarthritis International Society (ASAS) classification criteria [5] for the earlier detection of cases without radiographic sacroiliitis, a higher number of females have been identified as having axSpA. Though the male-to-female ratio of radiographic axSpA was 3:1 in the 1990s [6], newer cohorts have reported an equal prevalence, particularly for non-radiographic axSpA [2].

Different disease characteristics of axSpA between sexes have been recognized. In both AS and non-radiographic axSpA, women generally present with higher disease activity [7–10]. The axial skeleton, which is the primary site of involvement in axSpA, presents challenges in assessing disease activity compared to the peripheral joints, which are commonly affected in rheumatoid arthritis. Therefore, patient-reported outcome measures, such as the Bath Ankylosing Spondylitis Disease Activity Score (BASDAI) [11] are frequently used to evaluate disease activity in patients with axSpA. However, previous studies comparing pain-related patient-reported outcome measures between sexes have consistently reported higher scores in females with various diseases including axSpA [12–14]. In contrast, inflammatory laboratory values were significantly higher in males with axSpA [7–10]. Previous studies have reported similar disease activity scores between males and females when using composite measures such as the Ankylosing Spondylitis Disease Activity Score (ASDAS) [7, 8, 10], which combines patient-reported outcome measures with CRP [15], a type of inflammatory laboratory value.

In axSpA, non-steroidal anti-inflammatory drugs (NSAIDs) are the mainstay treatment, and for patients who do not respond, tumor necrosis factor alpha inhibitors (TNFi) can be considered a second-line therapy [16, 17]. Various recommendations [16–21] suggest utilizing disease activity scores, such as the BASDAI or ASDAS, as criteria for TNFi initiation and assessment of treatment efficacy. Despite the differences in the reporting of disease activity scores between sexes, the criteria for both males and females remained the same, which could potentially impact the assessment of medication efficacy. The Korean College of Rheumatology Biologics and Targeted Therapy (KOBIO) Registry is a prospective nationwide biological therapy registry that includes data from patients with axSpA. In this study, we aimed to investigate whether there are sex differences in disease activity scores among patients with axSpA using the KOBIO registry data and to determine any potential impact on the assessment of medication response.

## Methods

### Study design and data source

The data for this study were retrieved from the KOBIO Registry (ClinicalTrials.gov identifier NCT01965132) [22], a prospective nationwide registry for rheumatoid arthritis, axSpA, and psoriatic arthritis that includes 58 hospitals in South Korea. This registry enrolled patients with axSpA who started biologic disease modifying antirheumatic drugs with baseline clinical data and were followed up annually. Our target cohort population included patients (1) with axSpA who were enrolled in the registry between December 2012 and August 2021, (2) who were followed up at least once, and (3) who started TNFi treatment at baseline. All patients who met the modified New York criteria for AS or ASAS axial spondyloarthritis criteria and were older than 18 years were enrolled in the KOBIO registry. Patients lacking baseline clinical data or without assessments of their one-year treatment response were excluded from the study.

### Outcome variables

The following disease activity scores were included in the analysis: ASDAS (scored using CRP) [15], Patient Global Assessment of the Disease (PGA) [23], and BASDAI [11]. Sex was the primary variable of interest (main predictor).

Disease activity scores were assessed as baseline values upon enrollment in the KOBIO registry when the decision to initiate TNFi therapy was made. The KOBIO registry also conducts annual follow-ups and monitors the disease activity scores. If the TNFi treatment initiated at baseline was discontinued, the point of discontinuation was considered the first follow-up observation, regardless of whether it occurred before the scheduled annual follow-up.

### Confounding factors

Other variables of interest that were investigated as potential confounders included age, disease characteristics (disease duration, HLA-B27 positivity, and proportion of patients with non-radiographic axSpA), whether the patient was naïve to biologics, type of TNFi used, cigarette smoking, SpA features (presence of inflammatory back pain, presence of peripheral arthritis, enthesitis, uveitis, psoriasis, and Crohn’s disease at baseline, as well as family history of SpA), acute-phase reactants (erythrocyte sedimentation rate and CRP level), and comorbidities (hypertension, diabetes mellitus, ischemic heart disease, and chronic kidney disease).

### Impact of disease activity scores on the assessment of treatment outcome of TNFi therapy

Several criteria have been used to investigate the impact of differences in disease activity on the assessment of medication efficacy. In previous studies, TNFi efficacy has been assessed by either a reduction of 50% or more or a decrease of 2 or more in BASDAI (BASDAI-based criterion) [24, 25] or a reduction of 1.1 or more in ASDAS (ASDAS-based criterion) [16, 24]. Therefore, we analyzed the sex-specific differences in the proportion of patients showing improvement in disease activity who met these criteria at the first follow-up observation.

### Sensitivity analysis

If TNFi was discontinued for reasons other than inefficacy (e.g., adverse events, economic problems, or preparation for pregnancy), disease activity was not measured at the originally intended time point. To ensure the robustness of the study, a sensitivity analysis was performed by excluding drug discontinuation for reasons other than inefficacy.

### Statistical analysis

We analyzed the baseline characteristics and outcome measures at both the baseline and the first follow-up observation, focusing on identifying potential differences between sexes. The results were summarized as means and standard deviations (SD) for continuous variables and as frequency and percentage for categorical variables. Comparisons between sexes were performed using the chi-square test for categorical variables and the Wilcoxon rank-sum test or t-test for continuous variables, as appropriate. To investigate the relationship between changes in each outcome measure and sex, we conducted a multivariate linear regression analysis. Changes in outcome measures were defined as the difference between the values at the first follow-up observation and baseline. As covariates, we included baseline characteristics that exhibited significant differences between males and females, along with the baseline values of each outcome measure and the duration from baseline to the first follow-up observation for each individual. In addition, we performed an identical analysis using Inverse Probability of Treatment Weighting (IPTW) with the same covariates. IPTW involves two main steps. First, the probability, or propensity, of being exposed to the risk factor or intervention of interest is calculated, given an individual’s characteristics (i.e., propensity score). Second, weights are calculated as the inverse of the propensity score. The application of these weights to the study population creates a pseudopopulation in which confounders are equally distributed across exposed and unexposed groups [26]. We also conducted a multivariate logistic regression analysis and IPTW to analyze whether there were sex differences in the proportion of patients reaching the cutoff of disease activity scores related to medication response. Complete case analysis was used for missing data. For sensitivity analysis, we conducted a multivariate logistic regression analysis using identical covariates to evaluate the relationship between the outcome measures and sex.

## Results

### Baseline characteristics

This study included 1,753 patients with sufficient clinical data. The clinical characteristics of the patients with axSpA are shown in Table 1. The cohort showed a male predominance (*n* = 1,343, 76.6%) and the mean patient age was 39.69 (standard deviation [SD] 13.02) years. HLA-B27 positivity was observed in 89.7% of patients. A total of 1,583 (90.3%) patients had radiographic sacroiliitis that fulfilled the modified New York criteria for AS. Female patients were older, had a shorter disease duration, lower rates of HLA-B27 positivity, higher rates of non-radiographic SpA, higher incidence of peripheral manifestations, less frequent family history of axSpA, lower levels of CRP, and less frequent ischemic heart disease. The type of TNF inhibitor used was significantly different between sexes (*p* < 0.001), with infliximab was used at a higher rate in females compared to males. 79% of all patients were biologic-naïve, with no difference between sexes.

**Table 1:** Baseline characteristics of patients

	Total (*n* = 1,753)	Male (*n* = 1,343)	Female (*n* = 410)	*p*-value
Age, mean (SD), years	39.69 (13.02)	38.71 (12.75)	42.88 (13.40)	< 0.001
Disease duration, mean (SD), years	5.00 (6.17)	5.46 (6.36)	3.48 (5.22)	< 0.001
HLA-B27 positivity (*n* = 1,617) (%)	1451 (89.73)	1131 (91.88)	320 (82.9)	< 0.001
nr-axSpA (%)	170 (9.7)	118 (8.79)	52 (12.68)	0.020
Cigarette smoking (%)				< 0.001
No smoker	881 (50.26)	507 (37.75)	374 (91.22)	
Ex-smoker	365 (20.82)	351 (26.14)	14 (3.41)	
Current smoker	507 (28.92)	485 (36.11)	22 (5.37)	
ESR, mean (SD), mm/h	37.08 (29.93)	35.13 (29.12)	43.47 (31.63)	< 0.001
CRP (*n* = 1,733), mean (SD), mg/dL	2.14 (2.89)	2.23 (2.92)	1.85 (2.78)	< 0.001
Inflammatory back pain (*n* = 1,748) (%)	1475 (84.38)	1134 (84.69)	341 (83.37)	0.521
Peripheral arthritis (*n* = 1,735) (%)	492 (28.36)	341 (25.66)	151 (37.19)	< 0.001
Enthesitis (*n* = 1,738) (%)	278 (16)	197 (14.81)	81 (19.85)	0.015
Uveitis (*n* = 1,740) (%)	164 (9.43)	112 (8.41)	52 (12.75)	0.009
Psoriasis (*n* = 1,738) (%)	41 (2.36)	34 (2.56)	7 (1.72)	0.328
Crohn’s disease (*n* = 1,734) (%)	10 (0.58)	7 (0.53)	3 (0.74)	0.708
Family history of SpA (*n* = 1,721) (%)	196 (11.39)	138 (10.47)	58 (14.39)	0.030
Hypertension (%)	274 (15.63)	213 (15.86)	61 (14.88)	0.632
DM (%)	87 (4.96)	69 (5.14)	18 (4.39)	0.542
Ischemic heart disease (*n* = 1,752) (%)	21 (1.2)	20 (1.49)	1 (0.24)	0.039
CKD^a^ (%)	15 (0.86)	12 (0.89)	3 (0.73)	> 0.99
Type of TNFi^b^ (%)				< 0.001
Adalimumab	696 (39.7)	546 (40.66)	150 (36.59)	
Etanercept	273 (15.57)	214 (15.93)	59 (14.39)	
Golimumab	387 (22.08)	309 (23.01)	78 (19.02)	
Infliximab	397 (22.65)	274 (20.4)	123 (30)	
Number of biologic agents used previously (%)				0.474
0	1,385 (79.01)	1,053 (78.41)	332 (80.98)	
1	270 (15.4)	211 (15.71)	59 (14.39)	
More than two	98 (5.59)	79 (5.88)	19 (4.63)	
Time interval between baseline to first follow-up, mean (SD), months	11.13 ± 3.78	11.22 ± 3.74	10.84 ± 3.90	0.077

*SD* standard deviation, *HLA-B27* human leukocyte antigen B27, *nr-axSpA* non-radiographic axial spondyloarthritis, *ESR* erythrocyte sedimentation rate, *CRP* C-reactive protein, *DM* diabetes mellitus, *CKD* chronic kidney disease, *TNFi* tumor necrosis factor alpha inhibitor.

^a^Glomerular filtration rate ≤ 60 mL/min/1.73 m^2^.

^b^Including original drug and their biosimilar agents.

### Outcome measures at baseline and the first follow-up observation

At baseline, the mean BASDAI, ASDAS, and PGA scores were 5.98 (SD, 1.99), 3.6 (SD, 1.07), and 6.33 (SD, 2.1), respectively. BASDAI was higher in females (5.91 vs. 6.24, *p* = 0.002), while ASDAS and PGA showed no significant differences between males and females. In the first follow-up observation, the mean BASDAI, ASDAS, and PGA of all patients were 2.7 (SD, 2.09), 1.68 (SD, 1.02), and 3.2 (SD, 2.25), respectively. BASDAI remained higher in females (2.60 vs. 3.02, *p* < 0.001); however, PGA showed a different pattern compared with baseline, as it was higher in females (3.09 vs. 3.55, *p* < 0.001). Data for the disease activity measurements are shown in Table 2.

**Table 2:** Disease activity measures at baseline and the first follow-up observation

Outcome	Total (*n* = 1753)	Male (*n* = 1343)	Female (*n* = 410)	*p*-value
Baseline				
BASDAI, mean (SD)	5.98 (1.99)	5.91 (1.95)	6.24 (2.08)	0.002
ASDAS, mean (SD)	3.6 (1.07)	3.62 (1.05)	3.55 (1.12)	0.259
PGA, mean (SD)	6.33 (2.1)	6.29 (2.08)	6.48 (2.17)	0.110
CRP, mg/dL, mean (SD)	2.14 (2.89)	2.23 (2.92)	1.85 (2.78)	< 0.001
**Follow-up**				
BASDAI, mean (SD)	2.7 (2.09)	2.6 (2.04)	3.02 (2.22)	0.001
ASDAS, mean (SD)	1.68 (1.02)	1.67 (1.01)	1.69 (1.04)	0.966
PGA, mean (SD)	3.2 (2.25)	3.09 (2.2)	3.55 (2.37)	< 0.001
CRP, mg/dL, mean (SD)	0.54 (1.54)	0.58 (1.61)	0.42 (1.27)	< 0.001

*BASDAI* Bath Ankylosing Spondylitis Disease Activity Score, *SD* standard deviation, *ASDAS* Ankylosing Spondylitis Disease Activity Score, *CRP* C-reactive protein, *PGA* Patient Global Assessment of the Disease.

### Assessment of the impact of sex on the changes in outcome measures

To investigate the influence of sex on the changes in disease activity between baseline and the first follow-up, baseline characteristics that exhibited significant differences between sexes, baseline value of each outcome, and the time interval between baseline and the first follow-up were adjusted (Table 3). After adjusting for other variables using multivariate linear regression analysis, the changes in BASDAI and PGA were independently associated with sex (BASDAI, 𝛽 = 0.343, *p* = 0.011; PGA, 𝛽 = 0.491, *p* = 0.001; 𝛽>0 indicates that there were more significant changes in males), but the changes in ASDAS showed no significant association with sex (𝛽 = 0.079, *p* = 0.235). In the IPTW analysis with more stringent confounder correction, the association of sex with the changes of BASDAI and ASDAS showed consistent results (BASDAI, 𝛽 = 0.262, *p* = 0.028; ASDAS, 𝛽 = -0.011, *p* = 0.846). However, the significance of the association for PGA was lost.

**Table 3:** Multivariate linear regression analysis and inverse probability of treatment weighting (IPTW) of change in each outcome from baseline to the first follow-up by sex. All analyses corrected for variables^a^ that were significantly different between sexes in baseline characteristics, baseline value of each outcome, and the time interval between baseline to the first follow-up

Outcome	Multivariate linear regression analysis			IPTW		
	β^b^ (95% CI)	SE	*p*-value	β^b^	SE	*p*-value
BASDAI	0.343 (0.079–0.607)	0.135	0.011	0.262 (0.028–0.496)	0.119	0.028
ASDAS	0.079 (-0.501–0.209)	0.066	0.235	-0.011 (-0.121–0.099)	0.056	0.846
PGA	0.491 (0.208–0.775)	0.145	0.001	0.028 (-0.218–0.273)	0.125	0.823

*IPTW* inverse probability of treatment weighting, *CI* confidence interval, *SE* standard error, *BASDAI* Bath Ankylosing Spondylitis Disease Activity Score, *ASDAS* Ankylosing Spondylitis Disease Activity Score, *CRP* C-reactive protein, *PGA* Patient Global Assessment of Disease.

^a^Included variables were age, disease duration, HLA-B27 positivity, radiographic changes, cigarette smoking, peripheral arthritis, uveitis, family history of spondyloarthritis, enthesitis, and type of tumor necrosis factor alpha inhibitors.

^b^The regression coefficient was calculated for the change in the disease activity measure in males compared to females. A positive coefficient indicated a greater change in the disease activity measure in males, while a negative coefficient indicated a smaller change in the disease activity measure in males.

### The impact of sex differences in outcome measures on the assessment of treatment response

The BASDAI and ASDAS have been widely used as outcome measures to evaluate response to biological agents in patients with axSpA. Previously, we observed a significant relationship between sex and changes in the BASDAI, whereas ASDAS did not show a significant relationship. Therefore, we investigated whether these differences could potentially affect the evaluation of treatment response to biological agents using BASDAI-based and ASDAS-based criteria. Both criteria were judged to be effective treatment responses in approximately 70% of the patients at follow-up. There were no statistically significant differences in the proportion of effective treatment responses to TNFi between sexes using either criterion (Table 4). However, when adjusted for baseline characteristics, baseline value of each outcome, and follow-up interval, male patients were more likely to be assessed as effective by the BASDAI-based criterion (OR 1.700, 95% CI, 1.200–2.406; *p* = 0.003), whereas the ASDAS-based criterion showed no significant difference between sexes (OR 0.993, 95% CI, 0.678–1.455; *p* = 0.973; Table 5). Results for all variables used in the multivariate logistic regression are included in Supplementary Table 1. Consistent trends were observed in the IPTW analysis (Table 5).

**Table 4:** Comparison of differences between sexes when TNFi response was assessed using BASDAI and ASDAS

Outcome	Treatment responses	Total	Male	Female	*p*-value
BASDAI criterion		*n* = 1699	*n* = 1302	*n* = 397	0.147
	Responder	1213 (71.39)	941 (72.27)	272 (68.51)	
	Non-responder	486 (28.61)	361 (27.73)	125 (31.49)	
ASDAS criterion		*n* = 1653	*n* = 1262	*n* = 391	0.317
	Responder	1187 (71.81)	914 (72.42)	273 (69.82)	
	Non-responder	466 (28.19)	348 (27.58)	118 (30.18)	

Statistical comparisons were made using the Chi-square test.

*BASDAI* Bath Ankylosing Spondylitis Disease Activity Score, *ASDAS* Ankylosing Spondylitis Disease Activity Score.

BASDAI criterion: If BASDAI decreases by 50% or more of the baseline, or by two or more, the patient was classified as a responder.

ASDAS criterion: If ASDAS decreases by 1.1 or more of the baseline, the patient was classified as a responder.

**Table 5:** Comparisons of sex differences in TNFi response using BASDAI and ASDAS, adjusted for variables^a^ by multivariate logistic regression analysis and inverse probability of treatment weighting (IPTW)

Outcome	Multivariate logistic regression analysis		IPTW	
	OR^d^ (95% CI)	*p*-value	OR (95% CI)	*p*-value
BASDAI criterion^b^	1.700 (1.200–2.406)	0.003	1.388 (1.046–1.842)	0.023
ASDAS criterion^c^	0.993 (0.678–1.455)	0.973	0.890 (0.648–1.222)	0.471

*BASDAI* Bath Ankylosing Spondylitis Disease Activity Score, *ASDAS* Ankylosing Spondylitis Disease Activity Score, *IPTW* inverse probability of treatment weighting, *OR* Odds ratio.

^a^Included variables were age, disease duration, HLA-B27 positivity, radiographic changes, cigarette smoking, peripheral arthritis, uveitis, family history of spondyloarthritis, enthesitis, type of tumor necrosis factor alpha inhibitors, baseline value of each outcome, and the time interval between baseline to the first follow-up.

^b^BASDAI criterion: If BASDAI decreases by 50% or more of the baseline, or by two or more, the patient was classified as a responder.

^c^ASDAS criterion: If ASDAS decreases by 1.1 or more of the baseline, the patient was classified as a responder.

^d^The calculated odds ratio of reaching the response criteria in male patients compared to female patients. Odds ratios above 1.0 indicate that male patients are more likely to be found effective for each criterion, while odds ratios below 1.0 indicate that male patients are less likely to be found effective.

### Sensitivity analysis

Sensitivity analyses were performed, excluding patients who discontinued TNFi for reasons other than inefficacy. A total of 1,533 patients were analyzed. After adjusting for confounding variables using multivariate linear regression analysis, changes in BASDAI and PGA were independently associated with sex, but not with changes in ASDAS. In addition, male patients were more likely to be assessed as effective by the BASDAI-based criteria, whereas the ASDAS-based criteria did not differ significantly between sexes. The results were identical for all patients (Supplementary Tables 1 and 2).

## Discussion

We found that at the start of TNFi, BASDAI, a disease activity score consisting of only patient-reported outcomes, was higher in females than in males; however, the ASDAS, a combined disease activity score with CRP, did not differ between sexes. At follow-up, BASDAI was still higher in females, ASDAS did not differ between the sexes, and PGA was significantly lower in males compared to females. When comparing the changes between follow-ups, after adjusting for confounding factors, BASDAI improved significantly more in males, whereas ASDAS did not differ between sexes. These disparities also affect the assessment of actual TNFi treatment efficacy. When using the BASDAI-based criterion, there was a higher likelihood of determining a favorable TNFi treatment effect at follow-up in males compared to females, whereas use of the ASDAS-based criterion showed no significant difference in effectiveness assessment between sexes.

Differences in disease activity between sexes have been reported in patients with axSpA. Previous studies have also reported significant differences in BASDAI between males and females, similar to this study, whereas no significant differences were observed in ASDAS [12, 27, 28]. The specific reasons for variations in disease activity between men and women are not completely understood. First, there is a difference in susceptibility to pain. In both musculoskeletal and nonmusculoskeletal conditions, women typically indicate higher levels of pain intensity than men do [29]. Central pain sensitization has been suggested as a possible explanation for differences in axSpA pain between men and women. A classic example of pain arising from this mechanism is fibromyalgia, which can lead to higher reported pain and fatigue, and thus, higher reported disease activity. Fibromyalgia is more common in women than in men, with 89% of patients being women in the general population [30]. In patients with axSpA, fibromyalgia is also more prevalent in women, and there are reports that this may lead to an overestimation of disease activity in women [31]. In this study, it would have been valuable to compare the frequency of fibromyalgia between sexes. Unfortunately, the KOBIO registry does not collect fibromyalgia information; therefore, we could not perform this analysis. Second, different disease manifestations between sexes may have affected disease activity. In patients with axSpA, women have a relatively higher frequency of peripheral joint arthritis [27, 32] and enthesitis [7, 9, 27, 33] than men. In the BASDAI and ASDAS, the weight of each question was set differently. In BASDAI, the weight of the questions for both peripheral arthritis and enthesitis is set at 20%. However, in ASDAS, the weight for peripheral arthritis (tender and swollen joints) was set to approximately 10.4%, and there was no independent question for enthesitis [28]. Therefore, it is possible that BASDAI is more sensitive in distinguishing differences in peripheral manifestations than ASDAS. In a previous study, BASDAI was able to identify more peripheral manifestations in women, whereas ASDAS was unable to detect this difference [28]. In this study, both peripheral arthritis and enthesitis were significantly more frequent in women than in men, which may explain why there was a sex difference in the BASDAI at baseline but not in the ASDAS. However, when analyzing the change in disease activity from baseline to follow-up and whether a significant treatment effect was achieved based on BASDAI and ASDAS, linear regression analysis was performed with correction for disease manifestations that differed between men and women, including peripheral arthritis and enthesitis. Additionally, propensity score correction was applied, and there was a difference between men and women. Therefore, it could be due to sex differences rather than a difference due to disease manifestation.

This study used data from the KOBIO registry, which has the advantage of being a large multicenter study. All patients were enrolled at the time of biologic initiation. This allowed us to include a homogeneous population on a large scale. These results are similar to those of previous studies showing differences in disease activity between sexes; however, we found a similar trend at the time of TNFi initiation. We also found a significant difference in disease activity between men and women on the BASDAI during TNFi treatment and no significant difference in ASDAS between the sexes. Moreover, we also found that these differences were not only numerical but also made a difference in the proportion of patients reaching the threshold used to determine effectiveness in real-world practice, suggesting that the use of BASDAI or ASDAS may make a difference in clinical practice to determine TNFi effectiveness between men and women. It is also possible that the difference in disease activity was due to other factors, such as differences in demographics or disease manifestations between men and women with axSpA. However, this study included a sufficiently large number of patients that allowed propensity score correction; therefore, we found that the differences in BASDAI and ASDAS were independently caused by sex.

Historically, the BASDAI has been widely used as a clinical measure for assessing disease activity in axSpA. However, it is a fully patient-oriented metric that is not specific to the inflammatory response, does not reflect the importance of each variable, and does not account for redundancies between variables [34]. The ASDAS was developed more recently than the BASDAI; it adds blood test results for either CRP (ASDAS-CRP) or erythrocyte sedimentation rate (ESR, ASDAS-ESR), adds the PGA, and excludes fatigue and localized tenderness. The ASDAS is expected to be more objective than the BASDAI because it includes inflammatory markers. In addition, because fatigue and localized tenderness are excluded from the score, the ASDAS is less relevant to central sensitization or fibromyalgia than the BASDAI. For this reason, some reports have suggested that the ASDAS is better at measuring and classifying disease activity in patients with axSpA than the BASDAI [35, 36]. However, it is not clear how this difference affects real-world patient evaluations, and a previous study had reported no difference between the two metrics in treat-to-target therapy [37]. This study shows that there were differences in changes in disease activity measured by BASDAI and ASDAS between sexes in real-world settings and that these differences may lead to different judgments of treatment response between sexes in practice.

In this study, in addition to multivariate linear and logistic regression, we used the IPTW method to adjust for confounders. Given that this study is observational, comparing male and female patients who are expected to have significantly different demographic and disease characteristics, accurate adjustment of confounders is crucial. IPTW uses the propensity score to balance baseline patient characteristics in the exposed and unexposed groups by weighting each individual in the analysis by the inverse probability of their actual exposure [26]. The application of these weights to the study population creates a pseudopopulation in which confounders are equally distributed. The propensity score matching method is also frequently used to create a pseudopopulation using propensity scores. However, in this study, there were many unmatched patients when analyzed using matching methods due to significant differences in characteristics between males and females, resulting in substantial sample losses. Therefore, we decided to use IPTW, which is useful for adjusting confounding effects in observational studies. Its consistent results with multivariate regression analysis in this study further demonstrate effective adjustment for confounding factors.

This study has a few limitations. First, it did not include an analysis of the reasons for the differences in disease activity measures. Therefore, we do not know whether any differences between men and women or between the two disease activity measures are associated with the results of this study. Factors such as fibromyalgia could have influenced patient-reported outcomes and led to sex differences; however, we could not analyze such effects due to insufficient clinical information. Second, although we corrected all clinical indicators that were significantly different between the sexes, remnant bias may still exist. Third, there is no gold standard for measuring disease activity in patients with axSpA. Thus, it is difficult to determine whether the BASDAI or ASDAS is more related to a patient’s actual disease activity. However, the results of this study suggest that ASDAS is a more robust method that is less affected by sex. Finally, the KOBIO registry data utilized in this study targeted patients attending tertiary hospitals, potentially introducing selection bias. Caution is needed when extrapolating these findings to the general population.

## Conclusions

Disease activity changes before and after TNFi use were significantly different between male and female patients, as measured by the BASDAI, but not by the ASDAS. When judging the effectiveness of TNFi treatment, female patients were more likely than male patients to be judged as having insufficient treatment responses by BASDAI, while the results were similar for both sexes by ASDAS. This suggests that the effects of TNFi treatment may be interpreted differently between sexes depending on the disease activity measure used.

### Electronic supplementary material

Below is the link to the electronic supplementary material.

Supplementary Material 1

## Data Availability

No datasets were generated or analysed during the current study.

## Abbreviations

ASAS: Assessment in Spondyloarthritis International Society
ASDAS: Ankylosing Spondylitis Disease Activity Score
axSpA: axial spondyloarthritis
BASDAI: Bath Ankylosing Spondylitis Disease Activity Score
CKD: chronic kidney disease
CRP: C-reactive protein, AS:ankylosing spondylitis
DM: diabetes mellitus
ESR: erythrocyte sedimentation rate
HLA-B27: human leukocyte antigen B27
IPTW: Inverse Probability of Treatment Weighting
KOBIO: Korean College of Rheumatology Biologics and Targeted Therapy
NSAIDs: non-steroidal anti-inflammatory drugs
nr-axSpA: non-radiographic axial spondyloarthritis
OR: Odds ratio
PGA: Patient Global Assessment of the Disease
SD: standard deviations
SE: standard error
TNFi: tumor necrosis factor alpha inhibitors
