# Supplementary material for: Sex-specific disparities in disease activity scores among patients with axial spondyloarthritis and their implications for evaluating the response to tumor necrosis factor alpha inhibitor therapy
Source: Arthritis Res Ther. 2024 Apr 25;26:90. doi: 10.1186/s13075-024-03320-x (PMC11044537; doi:10.1186/s13075-024-03320-x)
Supplement: Supplementary file 1 — Supplementary Material 1 [file 13075_2024_3320_MOESM1_ESM.docx]

**Supplementary Table 1.** Comparisons of gender differences in TNFi response using BASDAI and ASDAS by multivariate logistic regression analysis (for all variables).

| **Outcome** | **Variable** | ***p*-value** | **Corrected *p*-value** | **OR (95% CI)** |
| --- | --- | --- | --- | --- |
| BASDAI criterion^a^ | Sex (reference: female) | 0.003 |  | 1.700 (1.200–2.406) |
|  | Age | 0.010 |  | 0.987 (0.976–0.997) |
|  | Disease duration | 0.126 |  | 0.983 (0.962–1.005) |
|  | HLA-B27 positivity | 0.091 |  | 1.42 (0.946–2.132) |
|  | Radiographic changes | 0.187 |  | 0.732 (0.460–1.164) |
|  | Smoking: overall | 0.002 |  | . |
|  | Ex-smoker | 0.022 | 0.044 | 0.654 (0.431–0.991) |
|  | Current smoker | <0.001 | <0.001 | 0.553 (0.379–0.808) |
|  | Peripheral arthritis | 0.761 |  | 1.048 (0.774–1.419) |
|  | Uveitis | 0.480 |  | 0.856 (0.555–1.320) |
|  | Family history of SpA | 0.103 |  | 0.721 (0.487–1.068) |
|  | Enthesitis | 0.166 |  | 0.779 (0.547–1.109) |
|  | Type of TNFi: overall | 0.060 |  | . |
|  | Etanercept vs. Adalimumab | 0.108 | 0.066 | 0.737 (0.468–1.160) |
|  | Golimumab vs. Adalimumab | 0.755 | 0.001 | 0.946 (0.616–1.452) |
|  | Infliximab vs. Adalimumab | 0.014 | >0.999 | 0.662 (0.443–0.989) |
|  | Baseline BASDAI | <0.001 |  | 1.604 (1.495–1.720) |
|  | Follow-up duration | <0.001 |  | 1.169 (1.130–1.209) |
| ASDAS criterion^b^ | Sex (reference: female) | 0.973 |  | 0.993 (0.678–1.455) |
|  | Age | 0.009 |  | 0.985 (0.974–0.996) |
|  | Disease duration | 0.179 |  | 0.983 (0.959–1.008) |
|  | HLA-B27 positivity | 0.204 |  | 1.337 (0.854–2.093) |
|  | Radiographic changes | 0.962 |  | 1.012 (0.630–1.624) |
|  | Smoking: overall | 0.116 |  | . |
|  | Ex-smoker | 0.310 | 0.619 | 0.812 (0.513–1.285) |
|  | Current smoker | 0.038 | 0.077 | 0.687 (0.457–1.031) |
|  | Peripheral arthritis | 0.222 |  | 1.241 (0.877–1.757) |
|  | Uveitis | 0.425 |  | 1.214 (0.754–1.956) |
|  | Family history of SpA | 0.072 |  | 0.673 (0.437–1.035) |
|  | Enthesitis | 0.941 |  | 1.016 (0.673–1.532) |
|  | Type of TNFi: overall | 0.110 |  | . |
|  | Etanercept vs. Adalimumab | 0.976 | 0.929 | 1.007 (0.603–1.679) |
|  | Golimumab vs. Adalimumab | 0.587 | 0.115 | 0.901 (0.570–1.425) |
|  | Infliximab vs. Adalimumab | 0.021 | 0.667 | 0.649 (0.415–1.016) |
|  | Baseline ASDAS | <0.001 |  | 4.205 (3.522–5.021) |
|  | Follow-up duration | <0.001 |  | 1.221 (1.173–1.270) |

*OR* Odds ratio, *BASDAI* Bath Ankylosing Spondylitis Disease Activity Score, *ASDAS* Ankylosing Spondylitis Disease Activity Score, *HLA* human leukocyte antigen, *SpA* spondyloarthritis, *TNFi* tumor necrosis factor alpha inhibitor

^a^BASDAI criterion: If BASDAI decreases by 50% or more of the baseline, or by two or more, the patient was classified as a responder

^b^ASDAS criterion: If ASDAS decreases by 1.1 or more of the baseline, the patient was classified as a responder
